# A Method for Real-Time Lung Nodule Instance Segmentation Using Deep Learning

**DOI:** 10.3390/life14091192

**Published:** 2024-09-20

**Authors:** Antonella Santone, Francesco Mercaldo, Luca Brunese

**Affiliations:** Department of Medicine and Health Sciences “Vincenzo Tiberio”, University of Molise, 86100 Campobasso, Italy; antonella.santone@unimol.it (A.S.); luca.brunese@unimol.it (L.B.)

**Keywords:** lung, nodule, cancer, adenocarcinoma, segmentation, object detection, classification, YOLO, deep learning, health

## Abstract

Lung screening is really crucial in the early detection and management of masses, with particular regard to cancer. Studies have shown that lung cancer screening, can reduce lung cancer mortality by 20–30% in high-risk populations. In recent times, the advent of deep learning, with particular regard to computer vision, demonstrated the ability to effectively detect and locate objects from video streams and also (medical) images. Considering these aspects, in this paper, we propose a method aimed to perform instance segmentation, i.e., by providing a mask for each lung mass instance detected, allowing for the identification of individual masses even if they overlap or are close to each other by classifying the detected masses into (generic) nodules, cancer or adenocarcinoma. In this paper, we considered the you-only-look-once model for lung nodule segmentation. An experimental analysis, performed on a set of real-world lung computed tomography images, demonstrated the effectiveness of the proposed method not only in the detection of lung masses but also in lung mass segmentation, thus providing a helpful way not only for radiologist to conduct automatic lung screening but also for discovering very small masses not easily recognizable to the naked eye and that may deserve attention. As a matter of fact, in the evaluation of a dataset composed of 3654 lung scans, the proposed method obtains an average precision of 0.757 and an average recall of 0.738 in the classification task. Additionally, it reaches an average mask precision of 0.75 and an average mask recall of 0.733. These results indicate that the proposed method is capable of not only classifying masses as nodules, cancer, and adenocarcinoma, but also effectively segmenting the areas, thereby performing instance segmentation.

## 1. Introduction

Lung cancer (LC) causes nearly 400,000 deaths annually in Europe (https://gco.iarc.fr/today/en, accessed on 19 June 2024). Unfortunately, most patients are diagnosed at an advanced stage, resulting in very low 5-year overall survival rates of less than 10% [1,2,3]. This is particularly tragic, as low-dose computed tomography (CT) screening has shown a survival benefit for a well-defined high-risk population since the National Lung Screening Trial (NLST) in 2011 [4,5,6], a finding later confirmed by several European randomized controlled trials [7,8,9]. Screening high-risk individuals could thus lead to earlier detection and significantly improve 5-year survival rates. However, the U.S. experience reveals challenges in implementing national screening programs, with less than 10% of at-risk individuals participating [10,11,12]. Similar difficulties are anticipated in other countries, suggesting that it may take years to achieve a stage shift and improved survival rates in cancer registries.

Another major opportunity to achieve earlier detection is through incidentally detected lung nodules, which must be distinguished from those found through screening. Incidental pulmonary nodules (IPNs) are a common finding in radiology, occurring on any CT scan that includes the lungs. With the increasing use of chest imaging for various reasons, the detection of IPNs is also rising. While only a small number of IPNs are early-stage LC, identifying and following up on these malignant nodules is crucial. The accurate assessment and appropriate follow-up of IPNs could be as vital as screening programs in reducing LC mortality.

This issue is particularly pressing since IPNs often occur in individuals who do not qualify for LC screening due to diverse sociodemographic and medical characteristics, forming a distinct group from the LC screening population. A major concern is that in the U.S., approximately 1.1 million IPNs annually are not properly followed-up with, as shown by several analyses of U.S. health data [13].

In recent years, specially designed IPN management programs have been developed, mainly in the U.S., to improve the follow-up process. Implementing these programs has increased the rate of early LC detection, especially among individuals ineligible for LC screening. Therefore, a combined approach that includes both LC screening for high-risk individuals and proper follow-up for those with IPNs is essential for reducing LC mortality across a large population of potentially affected individuals.

However, there is a lack of data on the real-world situation of IPNs in European countries. The reality of IPN management remains unclear, and we can only speculate on how many IPNs may be overlooked in Europe [13].

There are several important reasons to boost lung screening. First of all, early detection, i.e., screening, particularly with low-dose computed tomography (LDCT), allows for the detection of lung nodules at an earlier stage. Early detection is crucial because it increases the likelihood of successful treatment and improves survival rates. Moreover, screening can lead to a shift in diagnosis from advanced stages of lung cancer, which are harder to treat and have poorer prognoses, to earlier stages that are more amenable to curative treatment.

Screening can also help in reducing mortality; in fact, studies such as the National Lung Screening Trial (NLST) and European randomized controlled trials have demonstrated that LDCT screening in high-risk populations can significantly reduce lung cancer mortality. Furthermore, by identifying lung nodules early, screening can lead to earlier interventions, which can significantly improve 5-year overall survival rates.

With screening, it is also possible to detect high-risk populations: screening programs focus on individuals at high risk for lung cancer, such as long-term smokers and older adults, thereby maximizing the benefit-to-harm ratio of screening. The early detection of nodules in high-risk individuals allows also for prompt medical or surgical interventions, potentially preventing the progression to advanced lung cancer.

Screening programs often include regular follow-up scans to monitor identified nodules. This ensures that any changes in size or appearance that might indicate malignancy are promptly addressed. Systematic screening reduces the likelihood of incidental nodules being overlooked, ensuring that suspicious nodules are appropriately evaluated.

The early detection and treatment of lung cancer through screening can be more cost-effective than treating advanced-stage cancer. Early-stage treatments often involve less complex and less expensive interventions. Screening can help allocate healthcare resources more efficiently by reducing the need for extensive treatments associated with advanced-stage cancer.

There are also several psychological benefits from screening; for instance, for high-risk individuals, regular screening can provide reassurance and reduce anxiety about developing lung cancer. Early detection empowers patients with more treatment options and better-informed decisions about their health.

Unfortunately there are several barriers to screening campaigns, for instance, low participation rates; in fact, despite the benefits, participation in screening programs can be low, as seen in the U.S., where less than 10% of eligible individuals participate. Efforts to increase awareness and accessibility are needed. Screening can sometimes result in false positives, leading to unnecessary anxiety, further testing, and invasive procedures. Effective screening programs must balance the benefits of early detection with the risks of overdiagnosis and overtreatment.

Another problem is related to ensuring high-quality, standardized screening programs, which is essential for maximizing the benefits and minimizing the harms of lung cancer screening. Radiologists and healthcare providers need adequate training to accurately identify and manage lung nodules detected during screening.

In summary, the importance of screening in discovering lung nodules lies in its potential to detect lung cancer at an earlier, more treatable stage, thereby improving survival rates and reducing mortality. Despite the challenges, the benefits of systematic and well-implemented screening programs can be substantial for high-risk populations.

There are several kinds of masses that can be found in the lung: a mass in the lung is a larger, abnormal growth in the lung tissue, typically defined as being greater than 3 cm (about 1.2 inches) in diameter. Lung masses can be benign (non-cancerous) or malignant (cancerous), but they are more likely to be malignant compared to smaller lung nodules.

In detail, a lung nodule is a small, round, or oval-shaped growth in the lung, often detected incidentally during chest imaging tests such as X-rays or CT scans. It is typically less than 3 cm (about 1.2 inches) in diameter. Nodules larger than 3 cm are generally referred to as lung masses and are more likely to be malignant. They can be benign (non-cancerous) or malignant (cancerous). Causes of benign lung nodules include infections, inflammatory conditions, and non-cancerous growths. They require monitoring through follow-up imaging to determine if the nodules change in size or appearance, which can indicate malignancy.

A lung mass can be recognized as lung cancer if it is characterized by an uncontrolled growth of abnormal cells in the lung tissues, which can form tumors, interfere with normal lung function, and spread (metastasize) to other parts of the body.

The major types of lung cancer include non-small-cell lung cancer (NSCLC) and small-cell lung cancer (SCLC). Symptoms may include persistent cough, chest pain, shortness of breath, and unexplained weight loss. They can be diagnosed through imaging tests, biopsies, and other diagnostic procedures. Staging is based on the size and extent of the tumor, lymph node involvement, and presence of metastasis.

Adenocarcinoma is a subtype of non-small-cell lung cancer (NSCLC) that arises from glandular cells in the lungs, which are cells that secrete mucus and other substances. It represents the most common type of lung cancer, especially in non-smokers and women. It is often found in the outer regions of the lungs and tends to grow more slowly than other types of lung cancer, such as small-cell lung cancer. Symptoms are similar to other forms of lung cancer but may include more frequent mucus production due to the glandular origin of the cancer. Diagnosis and treatment are similar to other types of lung cancer, involving imaging, biopsy, and treatments such as surgery, radiation, chemotherapy, and targeted therapies.

The main difference is that a lung nodule is a small growth that can be either benign or malignant, while lung cancer specifically refers to malignant tumors that originate in the lung. Lung nodules are often detected incidentally and require monitoring to determine their nature, whereas lung cancer is diagnosed based on the presence of malignant cells and typically requires comprehensive treatment.

Lung cancer is a broad term encompassing various types of cancer that originate in the lung, including both non-small-cell lung cancer (NSCLC) and small-cell lung cancer (SCLC). Adenocarcinoma is a specific type of NSCLC that originates from glandular cells in the lung. Adenocarcinoma has distinct pathological and clinical features compared to other types of lung cancer, such as squamous-cell carcinoma or small-cell lung cancer.

In recent times, artificial intelligence (AI), with particular regard to computer vision, has demonstrated a great ability to discover disease automatically from medical images. This is the reason why researchers are working on the integration of computer vision models into medical diagnostics, considering the impact on the screening process, enhancing the accuracy, speed, and efficiency of healthcare delivery.

As a matter of fact, AI can be utilized to analyze medical images such as X-rays, CT scans, MRI, and mammograms with high precision. Machine learning algorithms, particularly deep learning algorithms, can identify patterns and anomalies that may not be easily detectable by human eyes.

Moreover, AI tools can assist radiologists by highlighting potential areas of concern in scans, such as tumors or fractures, thereby reducing the chance of oversight. Regarding pathology, digital pathology images can be analyzed to detect cancerous cells or other diseases, providing a second opinion to pathologists.

There are several tasks than can be automatized with the adoption of computer vision; one of these task is represented by so-called segmentation, i.e., a process that involves dividing an image into segments or regions that are easier to analyze and understand. This technique is fundamental for various applications such as object detection, recognition, and image classification. There are two main types of segmentation in computer vision: semantic segmentation and instance segmentation. Semantic segmentation assigns a label to each pixel in an image, categorizing them into predefined classes. Instance segmentation not only classifies each pixel but also distinguishes between different instances of the same class. For example, it can separate two different cars in the same image, labeling them as distinct instances.

With regard to the state-of-the-art methods in the literature on disease detection using AI, convolutional neural networks (CNNs) [14,15,16,17], deep convolutional neural networks (DCNNs) [18,19,20], and recurrent neural networks (RNNs) are the most commonly used learning algorithms in medical imaging [21]. The CNN architecture is highly favored for supervised deep learning tasks such as lesion segmentation and classification due to its minimal preprocessing requirements. Recently, CNNs have been utilized in medical imaging for tasks like image segmentation (e.g., Mask R-CNN [22]) and classification (e.g., AlexNet [23] and VGGNet [24]). DCNNs, which include more layers and complex nonlinear relationships, have demonstrated reasonable accuracy in classification and regression tasks [25,26]. RNNs, as higher-order neural networks, can re-input the network output, allowing them to capture and exploit cross-slice variations to incorporate volumetric patterns of nodules, but they are susceptible to the vanishing gradient problem [27].

In this paper, we propose a method aimed to detect and locate the presence of lung nodules, cancer, and adenocarcinoma from medical images, by considering instance segmentation.

As instance segmentation model, we consider the you-only-look-once model i.e., YOLO, in particular, YOLO version 8; as a matter of fact, YOLOv8 has demonstrated interesting performances in both object detection and instance segmentation, especially in speed and accuracy, making it suitable for real-time applications. Differently from state-of-the-art methods in the literature that are mainly focuses on classification, we focused on instance segmentation, i.e., a computer vision task relating to the identification and delineation of individual objects within an image while assigning a unique label to each pixel, differently from the classification that aims to assign a label to a full image. In the current state-of-the-art literature, there have been several attempts to exploit the YOLO model for lung nodule segmentation; for instance, the researchers in [28,29,30,31] adopted the YOLO model for lung nodule segmentation, but differently from the proposed method, they adopt previous versions of the YOLO model; in particular, some authors considered the YOLO5 version, while the authors in [29,30,31] proposed the adoption of YOLO7. Also, methods exploiting CNNs have been proposed by researchers [32,33]: in this case, the main differences with respect to the proposed method is represented by the fact that CNNs can mark an image with a related label, without the ability to perform instance segmentation (i.e., to locate and detect the nodule in the image under analysis).

The remainder of this paper proceeds as follows: in the next section, an overview of the state-of-the-art research in lung cancer segmentation is provided, Section 3 introduces the method we propose for lung nodule instance segmentation, Section 4 discusses the experimental analysis obtained through the application of the proposed method on a real-world dataset of medical images, and, in the last section, the conclusion and future research lines are presented.

## 2. Related Work

Lung segmentation is a critical task in medical image analysis, enabling the precise identification of lung regions for subsequent diagnostic or therapeutic processes. Over the past decade, deep learning has revolutionized this field by providing robust and automated solutions. This section reviews significant contributions in lung segmentation using deep learning, focusing on the methods, datasets, and performances reported in the literature.

With regard to the state-of-the-art literature on disease detection using AI, CNNs, deep convolutional neural networks (DCNNs), and recurrent neural networks (RNNs) are the most commonly used learning algorithms in medical imaging [21]. The CNN architecture is highly favored for supervised deep learning tasks such as lesion segmentation and classification due to its minimal preprocessing requirements. Recently, CNNs have been utilized in medical imaging for tasks like image segmentation (e.g., Mask R-CNN [22]) and classification (e.g., AlexNet [23] and VGGNet [24]). DCNNs, which include more layers and complex nonlinear relationships, have demonstrated reasonable accuracy in classification and regression tasks [25,26]. RNNs, as higher-order neural networks, can re-input the network output, allowing them to capture and exploit cross-slice variations to incorporate volumetric patterns of nodules, although they are susceptible to the vanishing gradient problem [27].

Reinforcement learning was first applied by Google DeepMind in 2013 [34], and since then, it has been extensively explored to enhance lung cancer detection accuracy, sensitivity, and specificity. Semi-supervised learning techniques, such as deep reinforcement learning and generative adversarial networks, utilize labeled datasets. Supervised learning involves using a learning algorithm, where labels are assigned to input data during training. Various supervised deep learning methods have been applied to CT images for identifying abnormalities with anatomical localization. However, these methods have limitations, including the need for large amounts of labeled data, fixed network weights after training, and no capacity for post-training improvement. To address this, few-shot learning (FSL) models [35,36] have been developed to reduce data requirements during training.

Several deep learning approaches have been explored for lung segmentation. Wang et al. [37] developed a multi-view CNN (MV-CNN) for lung nodule segmentation, achieving an average DSC of 77.67% and an average ASD of 0.24 on the LIDC-IDRI dataset. Unlike conventional CNNs, MV-CNN integrates multiple input images for lung nodule identification, though it struggles with 3D CT scans. Consequently, a 3D CNN was created to handle volumetric patterns of cancerous nodules [38]. Sun et al. [39] designed a two-stage CAD system for automatic lung nodule segmentation and false positive (FP) reduction. This system, tested on the LIDC-IDRI dataset and evaluated by four experienced radiologists, achieved an average F1_score of 0.8501 for lung nodule segmentation.

In 2020, Cao et al. [40] introduced a dual-branch residual network (DB-ResNet) that captures multi-view and multi-scale features of nodules simultaneously. DB-ResNet, evaluated on the LIDC-IDRI dataset, achieved a DSC of 82.74%, outperforming trained radiologists. In 2021, Banu et al. [41] proposed the attention-aware weight-excitation U-Net (AWEU-Net) architecture for lung nodule segmentation in CT images. This architecture includes two stages: lung nodule detection using a fine-tuned Faster R-CNN and lung nodule segmentation using U-Net with position and channel attention-aware weight excitation. AWEU-Net achieved DSCs of 89.79% and 90.35% and IoUs of 82.34% and 83.21% on the LUNA16 and LIDC-IDRI datasets, respectively. Dutta [42] developed Dense R2Unet, a dense recurrent residual CNN based on U-Net with dense interconnections, which demonstrated superior segmentation performance compared to U-Net and ResUNet on a lung segmentation dataset.

One of the pioneering works in this domain is by Ronneberger et al. [43], who introduced the U-Net architecture, a convolutional neural network designed for biomedical image segmentation. This architecture has been widely adopted and modified for lung segmentation tasks due to its ability to capture context and high-resolution features simultaneously.

Christ et al. [44] proposed a two-step approach involving a lung segmentation step followed by nodule detection. Their method utilizes a fully convolutional network (FCN) and showed promising results on the LUNA16 dataset, demonstrating the effectiveness of deep learning in handling medical images.

Jin et al. [45] developed a 3D U-Net model to leverage volumetric information in CT scans. Their approach improved the segmentation accuracy by considering the spatial coherence across slices. The results on the LUNA16 dataset indicated superior performance compared to traditional 2D methods.

Another significant contribution is by Hofmanninger et al. [46], who introduced a self-supervised learning approach to enhance lung segmentation. By pre-training their model on a large dataset with a self-supervised task, they achieved improved performance on the LIDC-IDRI dataset.

The authors of [47] presented a hybrid approach combining deep learning and traditional image processing techniques. Their method integrates a CNN with conditional random fields (CRFs) to refine the segmentation boundaries, yielding better delineation of lung regions on the JSRT dataset.

In recent years, attention mechanisms have been incorporated into segmentation networks to improve focus on relevant features. The researchers in [48] developed an attention-guided U-Net, which significantly enhanced segmentation accuracy on the COVID-19-CT-Seg dataset by emphasizing critical regions within the lung fields.

Another notable study by Zheng et al. [49] utilized a dual-branch network to separately process lung fields and lesions, achieving state-of-the-art results on the COVID-19-CT-Seg dataset. This approach allowed for better handling of heterogeneous appearances of lung lesions.

Chen et al. [50] introduced a transformer-based architecture for lung segmentation, leveraging the global context provided by transformers to enhance segmentation performance on the LUNA16 and COVID-19-CT-Seg datasets.

The authors of [51] proposed a cross-attention network to further refine the segmentation process. By integrating cross-attention mechanisms, their model achieved improved accuracy and robustness, particularly in challenging cases with severe pathologies.

Finally, Wang et al. [52] explored the use of generative adversarial networks (GANs) for lung segmentation. Their model, trained on the LUNA16 and LIDC-IDRI datasets, demonstrated high accuracy and generalizability by generating more realistic segmentations.

Table 1 summarizes state-of-the-art methods in lung segmentation using deep learning, including details on the datasets used and the performance metrics reported.

Advancements in deep learning have significantly enhanced the lung instance segmentation performance. State-of-the-art models, such as Mask R-CNN and U-Net-based architectures, have achieved impressive accuracy in segmenting individual lung structures from medical images. For instance, Mask R-CNN can reach average Intersection over Union (IoU) scores exceeding 85%, while U-Net models often achieve Dice coefficients above 90% for lung segmentation tasks.

Recent developments also involve Transformer-based architectures and attention mechanisms, which have further refined the segmentation accuracy. These techniques improve the model’s ability to distinguish between overlapping lung regions and small structures, often resulting in accuracy improvements of 3–5% over previous methods. Additionally, integrating multi-scale and multi-modal data has enhanced performance, with some models achieving Dice scores and IoU metrics that approach or exceed 95%, especially in complex cases involving lesions and abnormalities.

Overall, deep learning has made substantial strides in lung instance segmentation, leading to more accurate and robust models that contribute to better diagnostic and treatment outcomes in pulmonary medicine.

Differently from the cited works, in this paper, we propose a method aimed to detect and locate the presence of lung nodules, cancer, and adenocarcinoma from medical images, by considering instance segmentation.

## 3. Deep Learning for Lung Nodule Instance Segmentation

In this section, we present the proposed method for detecting and localizing objects in lung CT, with a specific focus on identifying (generic) nodules, cancer, and adenocarcinomas by exploiting deep learning.

**Figure 1:**
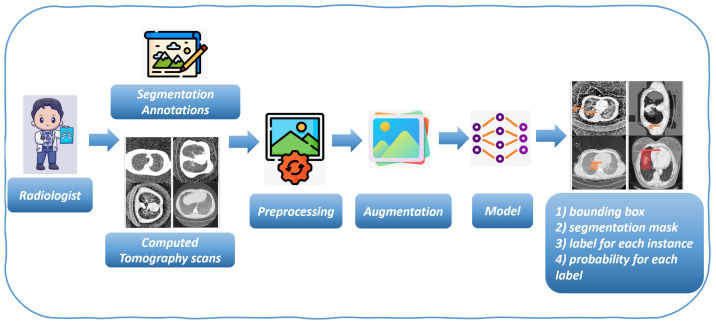
The proposed method.

Figure 1 demonstrates the proposed approach: alongside each detected nodule, the proposed method presents the prediction percentage, reflecting the confidence of the model in the detection accuracy.

To build an effective deep learning model for object detection in lung CT, we need a dataset consisting of CT related to lung. To create a model capable of identifying not only the presence of lung nodules but also their locations within the CT image, these images must be manually labeled by expert radiologists. This involves annotating the images with bounding boxes that mark the areas where the objects, specifically lung nodules, cancer and adenocarcinoma, are located (as shown in Figure 1).

To ensure that the model accurately predicts unseen lung CT images, the dataset should encompass images captured from diverse angles and under varying conditions. Despite initial differences in image sizes, a preprocessing step is necessary to resize all images to a uniform dimension.

The next step involves increasing the dataset size through image augmentation (depicted in Figure 1). This technique introduces controlled random modifications, such as rotations, flips, and trims, to the existing images, creating new variations. Data augmentation enhances the neural network’s ability to generalize by exposing it to a wider array of scenarios, thus reducing the risk of overfitting, where the model performs well on training data but poorly on unseen data.

In the follow, we further explain the techniques we considered for preprocessing and for augmentation: for each technique, we explain the purpose and the working mechanism.

Regarding preprocessing, we apply the following techniques:

- Auto-Orient
  - Purpose: Ensures that all images are oriented correctly.
  - How It Works: This technique reads the EXIF metadata of the image, which includes the orientation information (e.g., landscape, portrait, upside-down). It then rotates the image accordingly so that it is displayed correctly, regardless of how the photo was taken.
- Resize: Stretch to 640 × 640
  - Purpose: Standardizes the size of all images to 640 × 640 pixels.
  - How It Works: Each image is resized to fit within a 640 × 640 pixel frame. “Stretch” indicates that the aspect ratio is not preserved, so the image may be distorted to fit the specified dimensions.
- Auto-Adjust Contrast: Using histogram equalization.
  - Purpose: Enhances the contrast of the image.
  - How It Works: Histogram equalization is a method that adjusts the contrast of an image by redistributing the intensity values. It spreads out the most frequent intensity values, which can enhance the contrast of the image and make features more distinguishable.
- Grayscale
  - Purpose: Converts the image to grayscale.
  - How It Works: The image is converted from a color image (RGB) to a grayscale image where each pixel represents the intensity of light. This simplifies the image data and can reduce computational complexity while focusing on the structural information of the image.

Regarding augmentation, we applied following techniques:

- Outputs per training example: three.
  - Purpose: Generates multiple variations of each training example to increase the dataset’s size and diversity.
  - How It Works: For each original image, three different augmented versions are created using the specified augmentation techniques. This helps the model generalize better by exposing it to a wider variety of image transformations.
- Rotation: Between −10° and +10°.
  - Purpose: Introduces variability in the orientation of the images.
  - How It Works: Each image is randomly rotated by an angle between −10 degrees and +10 degrees. This helps the model become invariant to small rotations and improves its ability to recognize objects from different angles.
- Hue: Between −15° and +15°
  - Purpose: Adjusts the color tone of the image.
  - How It Works: The hue of the image is randomly adjusted within the specified range. This means that the colors in the image will shift slightly, helping the model learn to recognize objects regardless of slight changes in color.
- Saturation: Between −25% and +25%.
  - Purpose: Varies the intensity of colors in the image.
  - How It Works: The saturation level of the image is randomly increased or decreased by up to 25%. This means that the colors can become more vibrant or more muted, aiding the model in recognizing objects with different color intensities.
- Brightness: Between −10% and +10%.
  - Purpose: Adjusts the brightness of the image.
  - How It Works: The brightness of the image is randomly altered within the specified range. This means that the image can become slightly brighter or darker, helping the model handle varying lighting conditions.
- Exposure: Between −15% and +15%.
  - Purpose: Simulates changes in exposure.
  - How It Works: The exposure of the image is randomly adjusted by up to ±15%. This affects the overall lightness or darkness of the image, helping the model learn to recognize features under different exposure levels.

These preprocessing and augmentation techniques work together to prepare the images for training in a way that enhances model performance. Preprocessing steps ensure that all images are standardized and have enhanced contrast, while augmentation techniques create variations of the images to make the model robust to changes in orientation, color, and lighting. This combination helps in building a more generalizable and resilient model.

After preparing the augmented images with their corresponding annotations (bounding boxes for each generic nodule, cancer, and adenocarcinoma), we need a deep learning model for object detection. In this paper, we resort to the YOLO model [53], introduced by J. Redmon et al. in 2016; YOLO is the first one-stage object detection model, designed to classify images and detect object locations simultaneously.

YOLO’s primary advantage over other models is its speed. It processes the entire image in a single pass by dividing it into an SxS grid, where each grid cell is responsible for detecting an object if the object’s center falls within the cell. This single-stage detection pipeline enables YOLO to predict bounding boxes, object classes, and probabilities simultaneously, making it much faster than multi-stage models.

Although YOLO tends to make more localization errors compared to other models, it is less likely to produce false positives in the background and remains one of the fastest and most reliable models for object detection. In this paper, we consider the YOLOv8s version using the PyTorch framework.

The YOLO network consists of three main components: the backbone, the neck, and the head. The backbone, a CNN, extracts image features at various granularities. The neck combines these features to prepare them for the head, which makes the final predictions of bounding boxes and object classes.

As explained, the YOLO model is primarily designed for object detection rather than segmentation tasks. However, understanding how YOLO works for object detection helps in grasping how adaptations can be made for segmentation tasks. Below, we explain the working of YOLO for object detection and how similar principles can be applied to segmentation, followed by an explanation of the YOLO architecture.

YOLO is designed to detect objects in an image and simultaneously predict bounding boxes and class probabilities. In the following, we explain how it works:

- Grid Division: YOLO divides the input image into an SxS grid.
- Bounding Box Prediction: Each grid cell predicts a fixed number of bounding boxes. For each bounding box, it predicts the following:
  - The coordinates (x, y) of the box center relative to the grid cell.
  - The width and height of the box relative to the entire image.
  - The confidence score indicating the likelihood of the box containing an object and the accuracy of the box’s location.
- Class Prediction: Each grid cell also predicts the probabilities of each class for the objects it contains.
- Non-Maximum Suppression (NMS): YOLO applies NMS to filter out overlapping boxes, keeping only the most confident

While YOLO is not inherently designed for segmentation, it can be adapted for simple forms of segmentation by modifying the output to predict masks or contours of objects instead of just bounding boxes. This involves adding layers to predict a binary mask for each grid cell indicating the presence of an object.

The YOLO architecture consists of three main components: the backbone, the neck, and the head [54,55,56,57,58,59]:

1. Backbone: The backbone is a CNN used to extract features from the input image. Common backbones include versions of Darknet (e.g., Darknet-53) for YOLO. The backbone’s purpose is to create a feature map that highlights important features in the image. In this paper, the backbone was obtained via transfer learning using the imagenet dataset.
2. Neck: The neck is a series of layers that further process the feature map from the backbone. This typically involves layers like convolutional layers, upsampling, and feature concatenation to ensure that the network captures multi-scale features.
3. Feature Pyramid Network (FPN): Often used to improve the model’s ability to detect objects at different scales by combining low-resolution, semantically strong features with high-resolution, semantically weak features.
4. Head: The head is responsible for making the final predictions. It outputs a tensor that contains the bounding box coordinates, object confidence scores, and class probabilities for each grid cell.

Below, we list the processing pipeline of the YOLO object detection model:

1. Image Input: An image is input into the model and resized to a standard dimension.
2. Feature Extraction (Backbone): The image is passed through the backbone CNN, which extracts feature maps.
3. Feature Processing (Neck): The feature maps are processed through the neck, enhancing multi-scale feature representation.
4. Prediction (Head): The processed feature maps are passed through the head, where the following are performed:
  - Bounding box coordinates are predicted.
  - Object confidence scores are generated.
  - Class probabilities are calculated.
5. Postprocessing: Non-maximum suppression (NMS) is applied to remove duplicate detections, retaining the most confident predictions.

To adapt YOLO for segmentation tasks, the following modifications can be made:

1. Mask Prediction: Instead of just predicting bounding boxes, additional layers can be added to predict segmentation masks. Each grid cell can output a binary mask that indicates the presence of the object.
2. Loss Function: The loss function can be modified to include segmentation mask loss, in addition to the bounding box and classification loss.
3. Architectural Adjustments: The network architecture can be adjusted to include upsampling layers and skip connections similar to those used in fully convolutional networks (FCNs) or U-Net, which are common in segmentation tasks.

In detail, YOLO computes loss using three main components: localization loss, confidence loss, and classification loss. Below is the mathematical formulation of the YOLO loss function.

Relating the YOLO Loss Function, the total loss L in YOLO is a combination of three types of losses:

- Localization loss (bounding box regression loss);
- Confidence loss (objectness loss);
- Classification loss.

The combined loss functions can be expressed as

$$L=\lambda_{\mathrm{coord}}\sum_{i=0}^{S^{2}}\sum_{j=0}^{B}{⊮}_{ij}^{\mathrm{obj}}\left[{\left(x_{i}-{\hat{x}}_{i}\right)}^{2}+{\left(y_{i}-{\hat{y}}_{i}\right)}^{2}\right]+\lambda_{\mathrm{coord}}\sum_{i=0}^{S^{2}}\sum_{j=0}^{B}{⊮}_{ij}^{\mathrm{obj}}\left[{\left(\sqrt{w_{i}}-\sqrt{{\hat{w}}_{i}}\right)}^{2}+{\left(\sqrt{h_{i}}-\sqrt{{\hat{h}}_{i}}\right)}^{2}\right]$$

$$+\sum_{i=0}^{S^{2}}\sum_{j=0}^{B}{⊮}_{ij}^{\mathrm{obj}}{\left(C_{i}-{\hat{C}}_{i}\right)}^{2}+\lambda_{\mathrm{noobj}}\sum_{i=0}^{S^{2}}\sum_{j=0}^{B}{⊮}_{ij}^{\mathrm{noobj}}{\left(C_{i}-{\hat{C}}_{i}\right)}^{2}+\sum_{i=0}^{S^{2}}{⊮}_{i}^{\mathrm{obj}}\sum_{c\in \mathrm{classes}}{\left(p_{i}\left(c\right)-{\hat{p}}_{i}\left(c\right)\right)}^{2}$$

where −S×S is the grid size. −*B* is the number of bounding boxes per grid cell. −${⊮}_{ij}^{\mathrm{obj}}$ is an indicator function that equals 1 if the *j*-th bounding box in cell *i* is responsible for the prediction. −${⊮}_{ij}^{\mathrm{noobj}}$ is an indicator function that equals 1 if no object is present in the *j*-th bounding box in cell *i*. −$x_{i},y_{i}$ are the coordinates of the center of the bounding box. −${\hat{x}}_{i},{\hat{y}}_{i}$ are the ground truth coordinates of the center of the bounding box. −$w_{i},h_{i}$ are the width and height of the bounding box. −${\hat{w}}_{i},{\hat{h}}_{i}$ are the ground truth width and height of the bounding box. −$C_{i}$ is the confidence score for the bounding box. −${\hat{C}}_{i}$ is the ground truth confidence score. −$p_{i}\left(c\right)$ is the predicted probability of class *c*. −${\hat{p}}_{i}\left(c\right)$ is the ground truth probability of class *c*. −$\lambda_{\mathrm{coord}}$ and $\lambda_{\mathrm{noobj}}$ are the weights for the localization loss and confidence loss for cells without objects, respectively.

In the following, we explain the components of the loss function:

- Localization Loss (Bounding Box Regression Loss): This term penalizes errors in the predicted bounding box coordinates and dimensions.
  $$\lambda_{\mathrm{coord}}\sum_{i=0}^{S^{2}}\sum_{j=0}^{B}{⊮}_{ij}^{\mathrm{obj}}\left[{\left(x_{i}-{\hat{x}}_{i}\right)}^{2}+{\left(y_{i}-{\hat{y}}_{i}\right)}^{2}+{\left(\sqrt{w_{i}}-\sqrt{{\hat{w}}_{i}}\right)}^{2}+{\left(\sqrt{h_{i}}-\sqrt{{\hat{h}}_{i}}\right)}^{2}\right]$$
- Confidence Loss (Objectness Loss): This term penalizes errors in the confidence score, which reflects whether an object is present in the predicted bounding box.
  $$\sum_{i=0}^{S^{2}}\sum_{j=0}^{B}{⊮}_{ij}^{\mathrm{obj}}{\left(C_{i}-{\hat{C}}_{i}\right)}^{2}+\lambda_{\mathrm{noobj}}\sum_{i=0}^{S^{2}}\sum_{j=0}^{B}{⊮}_{ij}^{\mathrm{noobj}}{\left(C_{i}-{\hat{C}}_{i}\right)}^{2}$$
- Classification Loss: This term penalizes errors in the predicted class probabilities.
  $$\sum_{i=0}^{S^{2}}{⊮}_{i}^{\mathrm{obj}}\sum_{c\in \mathrm{classes}}{\left(p_{i}\left(c\right)-{\hat{p}}_{i}\left(c\right)\right)}^{2}$$

The overall loss function balances these components to train a model that accurately predicts bounding box coordinates, confidence scores, and class probabilities.

In the following, we describe the introduced notation:

- ${⊮}_{ij}^{\mathrm{obj}}$: indicator function for bounding boxes that contain objects.
- ${⊮}_{ij}^{\mathrm{noobj}}$: indicator function for bounding boxes that do not contain objects.
- $\lambda_{\mathrm{coord}}$: weight for localization loss, often set higher to emphasize precise localization.
- $\lambda_{\mathrm{noobj}}$: weight for confidence loss for cells without objects, usually set lower to reduce the impact of false positives.

These components together ensure that the YOLO model learns to predict bounding boxes accurately, with high confidence and correct classification.

While YOLO is primarily an object detection model, its architecture and principles can be adapted for segmentation by incorporating mask prediction and appropriate architectural modifications. The backbone extracts features, the neck processes these features, and the head makes predictions, which can be extended to include segmentation masks for each detected object.

Below, we provide a more technical explanation related to the steps needed to adapt the YOLO model for segmentation tasks; in particular, the modifications belong to the following categories: architectural, training, and postprocessing enhancements.

Below, we describe the architectural modifications:

- Mask Prediction Head
  - Addition: An additional head is introduced to predict the segmentation masks for each detected object. This head works alongside the existing heads responsible for bounding box regression and class prediction.
  - Structure: The mask head typically includes convolutional layers designed to output a binary mask for each detected instance. Each pixel in the mask indicates whether it belongs to the object or the background.
- Feature Pyramid Network (FPN) Enhancements
  - Purpose: FPNs are crucial for handling objects at various scales by effectively combining features from different layers of the backbone network.
  - Implementation: YOLOv8 leverages enhanced FPNs to ensure that both the detection and segmentation tasks benefit from rich, multi-scale feature representations.
- ROIAlign (Region of Interest Alignment)
  - Role: ROIAlign is used to extract fixed-size feature maps for each predicted bounding box. This technique ensures precise spatial alignment, which is critical for accurate mask predictions.
  - Functionality: Unlike ROI pooling, ROIAlign avoids quantization errors, using bilinear interpolation to compute the feature values, thereby preserving the spatial structure of the objects.
- Multi-Task Learning
  - Framework: YOLOv8 is trained in a multi-task learning setup where the backbone and neck networks are shared between the detection and segmentation tasks.
  - Benefits: This shared learning helps the model to learn more robust and generalizable features, improving performance on both tasks simultaneously.

In the following, we describe the training modifications:

- Segmentation Loss Function
  - Loss Addition: A segmentation loss, often a binary cross-entropy loss or a variant like Dice loss, is added to the overall loss function to train the mask head.
  - Purpose: This loss ensures that the predicted masks closely match the ground truth masks, optimizing the model for accurate instance segmentation.
- Data Preprocessing and Augmentation
  - Preprocessing: Images are resized and normalized to ensure consistency and efficiency during training.
  - Augmentation: Techniques such as random cropping, flipping, rotation, and color jittering are applied to increase the diversity of the training data and improve model generalization. Specific augmentations like CutMix and MixUp might also be used to help the model learn more robustly from partial object views and blended images.
- Anchor-Free Mechanism
  - Adaptation: For segmentation, the anchor-free mechanism allows the model to predict object locations without relying on predefined anchor boxes. This flexibility helps in dealing with varying object shapes and sizes more effectively.

In the following, we describe the last category, i.e., the postprocessing enhancements:

- Non-Maximum Suppression (NMS) for Masks
  - Application: NMS is applied to the predicted masks to eliminate redundant and overlapping predictions, ensuring that only the most confident and non-overlapping masks are retained.
  - Thresholding: Specific thresholds are set for the IoU (Intersection over Union) to filter out masks with high overlap, improving the final segmentation results.
- Mask Refinement
  - Techniques: Additional refinement steps, such as conditional random fields (CRFs) or simple morphological operations, may be used to smooth the predicted masks and correct any minor inaccuracies.

By incorporating these modifications, YOLOv8 is adapted for instance segmentation tasks. The model retains its efficiency and speed while achieving high accuracy in predicting both bounding boxes and detailed object masks, as shown in Section 4.

## 4. Experimental Analysis

In this section, we present the results of the experimental analysis aimed to demonstrate the effectiveness of the proposed method.

Regarding model training and testing, we exploited the Python programming language, in particular the 3.10 version. (https://www.python.org/, accessed on 19 June 2024) and PyTorch 2.4 version (https://pytorch.org/, accessed on 19 June 2024), a machine learning library based on the Torch library, used for applications such as computer vision and natural language processing. For model training, we utilized a machine equipped with an NVIDIA Tesla T4 GPU card featuring 16 GB of memory.

For our purposes, we considered the Lung Nodule Segmentation study Image Dataset [60], freely available for research purposes (https://universe.roboflow.com/varun-18tlk/lung-nodule-segmentation-study/dataset/3, accessed on 19 June 2024). The exploited dataset is composed of 3654 different images related to lung CT, across three classes: adenocarcinoma, cancer, and nodules. For each lung CT, there is a related segmentation annotation. The annotation process was performed by expert radiologists. The dataset is composed of 1650 images. On these images, we applied the following preprocessing techniques:

- Auto-Orient;
- Resize: stretch to 640 × 640;
- Auto-Adjust Contrast: using histogram equalization;
- Grayscale: applied.

Moreover, the following augmentation techniques were used to enrich the considered dataset:

- Rotation: between −10° and +10°;
- Hue: between −15° and +15°;
- Saturation: between −25% and +25%;
- Brightness: between −10% and +10%;
- Exposure: between −15% and +15%.

By applying these augmentation techniques for each image, we obtained three different images, so the final dataset was composed of 3654 images. We split the images in the following way: 3006 images for training (82%), 324 (9%) for validation, and the remaining 324 (9%) for testing.

**Figure 2:**
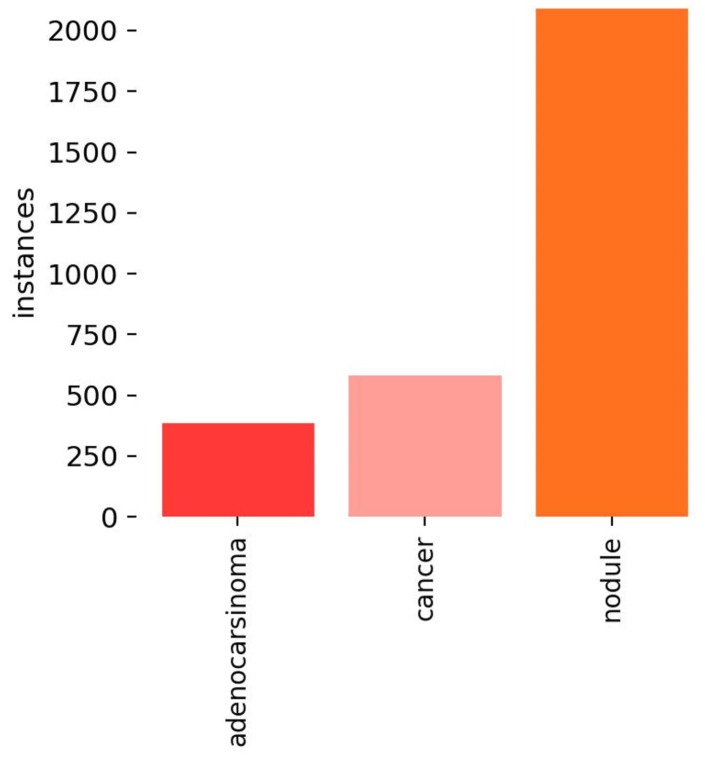
The number of instances for each category, i.e., nodule, cancer, and adenocarcinoma: it appears that in the dataset used, the numbers of instances for cancer and adenocarcinoma are quite similar, whereas the number of nodules is significantly higher compared to the other two categories.

Figure 2 shows the number of instances related to the analyzed dataset.

In detail, from Figure 2, it emerges that in the exploited dataset, the numbers of instances related to cancer and adenocarcinoma are quite similar, while the number of nodules is greater if compared with the remaining two categories.

**Figure 3:**
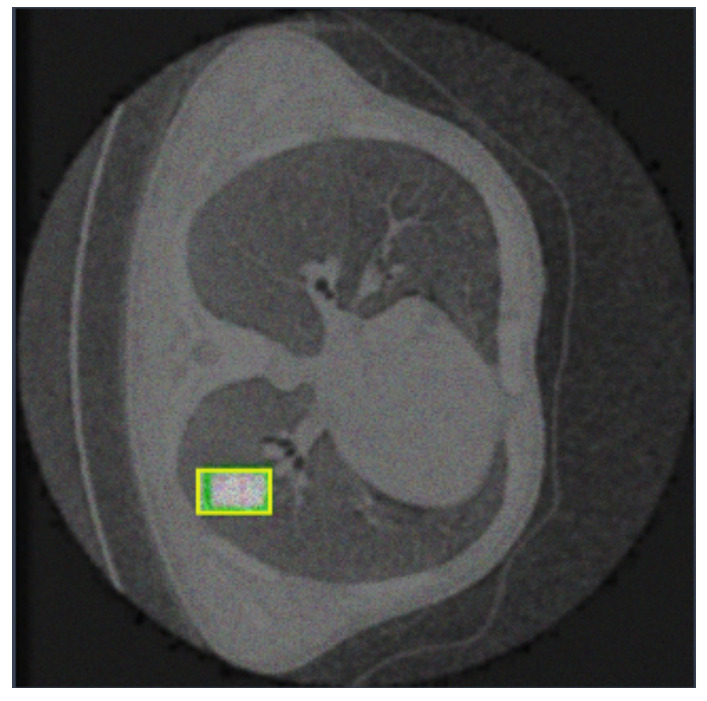
An example of a CT image belonging to the analyzed dataset, with the annotation related to a nodule.

**Figure 4:**
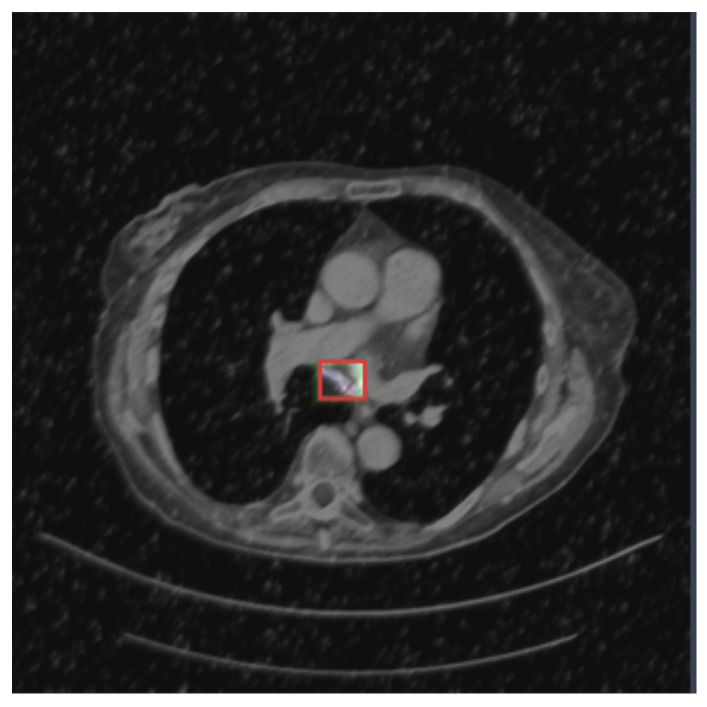
An example of a CT image belonging to the analyzed dataset, with the annotation related to lung cancer.

**Figure 5:**
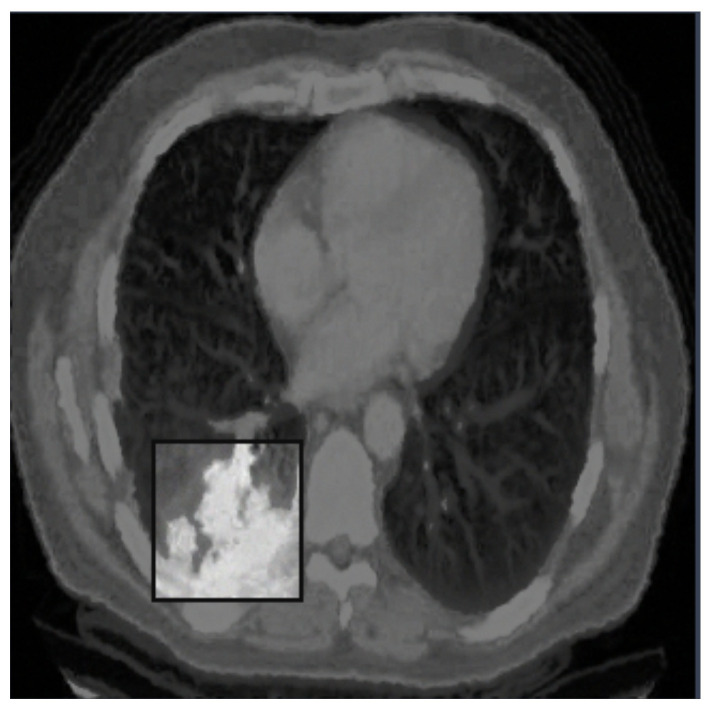
An example of a CT image belonging to the analyzed dataset, with the annotation related to adenocarcinoma.

Figure 3, Figure 4 and Figure 5 show three examples of lung CT belonging to the exploited dataset with the related segmentation annotation. In particular, in Figure 3, there is the segmentation annotation related to a nodule; in Figure 4, the segmentation annotation related to a cancer; and in Figure 5, the segmentation annotation related to an adenocarcinoma.

Regarding the model parameters, we used a batch size of 16, set the number of epochs to 50, and initialized the learning rate at 0.01. For model training, we utilized a machine equipped with an NVIDIA Tesla T4 GPU card with 16 GB of memory.

**Figure 6:**
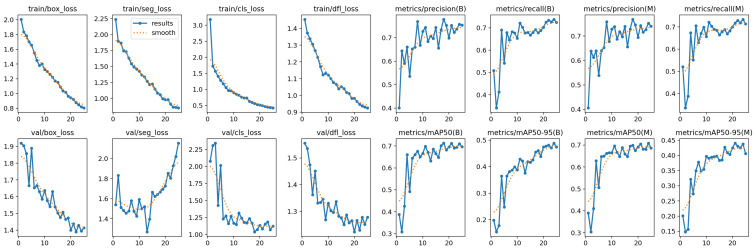
The results of the experimental analysis: The analyzed metrics exhibit the expected trends, i.e., precision, recall, mAP_0.5, and mAP_0.5:0.95 all increase with the number of epochs, indicating that the model is effectively improving its ability to detect objects in CT lung images.

In Figure 6, we show the experimental analysis results.

In the following, we describe the metrics related to each subplot in Figure 6. In the first line of the plots in Figure 6, we have the train/box_loss (i.e., the box_loss trend during the training: a loss that measures how “tight” the predicted bounding boxes are to the ground truth object); the train/seg_loss, referring to the segmentation loss during the training phase of a machine learning model (this loss measures the discrepancy between the predicted segmentation masks and the ground truth masks); the train/cls_loss trend (i.e., the cls_loss trend during the training: the cls_loss is a loss that measures the correctness of the classification of each predicted bounding box; each box may contain an object class or a ”background”; this loss is usually called cross-entropy loss); the metrics/precision(B), and the metrics/recall(B), the metrics/precision(M), and the metrics/recall(M), which are, respectively, related to the precision and recall for B and M, where (M) refers to “macro” average, which computes the average performance metric over each class, and (B) refers to the “best” performance metric achieved by the model during the training process.

The distribution focal loss (DFL) is a loss function designed to improve the training of object detection models by refining the predictions of bounding box coordinates. It is particularly used in methods like Generalized Focal Loss (GFL) for dense object detection, which aims to enhance the localization precision by focusing on the distribution of the predicted bounding boxes.

The distribution focal loss improves the localization of object bounding boxes by refining the predictions of box coordinates.

Thus, to formulate the DFL from a mathematical point of view, let us define the following: *y* is the ground truth value. *p* is the predicted value. cls(i) is the classification probability for the *i*-th bin. bin(i) is the center of the *i*-th bin. δ is the predicted distribution (logits or probabilities). σ(·) is the sigmoid function.

The distribution focal loss is defined as

$$L_{\mathrm{DFL}}=-\sum_{i=1}^{k}\left[y_{i}\log\left(\sigma \left(\delta_{i}\right)\right)+\left(1-y_{i}\right)\log\left(1-\sigma \left(\delta_{i}\right)\right)\right]$$

where *k* is the number of bins. $y_{i}$ is the ground truth distribution for the *i*-th bin. $\delta_{i}$ is the predicted distribution (logits or probabilities) for the *i*-th bin.

In the following, we explain the DFL formula:

- Ground Truth Value (*y*): This represents the true value of the bounding box coordinate. In the context of the DFL, this is usually expressed in terms of a probability distribution over a set of discrete bins.
- Predicted Value (*p*): This represents the predicted probability distribution over the bins for a bounding box coordinate.
- Classification Probability (cls(i)): This is the predicted probability for the *i*-th bin. It represents the likelihood that the bounding box coordinate falls within that bin.
- Bin Center (bin(i)): The center value of the *i*-th bin. Bins are used to discretize the continuous range of possible bounding box coordinates.
- Predicted Distribution (δ): The predicted logits or probabilities for each bin. These values are usually converted to probabilities using the sigmoid function.
- Sigmoid Function (σ(·)): This function is used to convert logits into probabilities. It ensures that the predicted values are between 0 and 1, suitable for probability distribution.

In practice, the distribution focal loss is combined with other losses, such as classification loss and confidence loss, to form the total loss function used to train the object detection model. The combined loss function can be expressed as

$$L=\lambda_{\mathrm{coord}}L_{\mathrm{coord}}+L_{\mathrm{conf}}+L_{\mathrm{cls}}+L_{\mathrm{DFL}}$$

where $L_{\mathrm{coord}}$ is the localization (bounding box regression) loss. $L_{\mathrm{conf}}$ is the confidence (objectness) loss. $L_{\mathrm{cls}}$ is the classification loss. $L_{\mathrm{DFL}}$ is the distribution focal loss. $\lambda_{\mathrm{coord}}$ is a weighting factor for the localization loss.

The distribution focal loss enhances the precision of bounding box predictions by focusing on the fine-grained distribution of the coordinates, leading to better localization performance in object detection models.

In the second line of the plots in Figure 6, we have the val/box_loss (i.e., the trend in the box_loss in the validation), *the val/seg_loss (i.e., the trend in the seg_loss in validation)*, val/cls_loss (aimed to quantiff the classification error of the predicted labels), and the val/dfl_loss (aimed to measure the discrepancy between the predicted and actual feature locations in the convolutional feature maps. The DFL, or distribution focal loss, is designed to tackle class imbalance in object detection across different categories. The “cls” value refers to the classification loss, which is computed using the Cross Entropy Loss function. Each of these losses is computed independently and then summed to form the final loss).

Metrics/mAP50(B) is the best average precision when the Intersection over Union is equal to 0.5 (mAP_0.5), metrics/mAP50(M) is the mean average precision when the Intersection over Union is equal to 0.5 (mAP_0.5), and metrics/mAP50-95(M) is the mean average precision when the Intersection over Union is from 0.5 to 0.95 (mAP_50-95).

All the metrics show the anticipated trends: the precision, recall, mAP_0.5, and mAP_0.5:0.95 increase with the number of epochs, indicating that the model is effectively learning to detect objects in CT lung images. Conversely, other metrics decrease as the number of epochs increases, further confirming the model’s correct learning process. The loss metrics, which represent the model’s errors in recognizing specific objects, are generally high in the initial epochs but decrease as the model enhances its detection capabilities over time.

Below are more details about the precision, recall, mAP_0.5, and mAP_0.5:0.95 metrics.

Precision measures the proportion of positive predictions that are correct, accounting for false positives, which are cases incorrectly flagged for inclusion. It can be calculated as follows:

$$Precision=\frac{TP}{TP+FP}$$

From the precision trend shown in Figure 6, we observe that precision increases over the epochs. This upward trend indicates that the network is effectively learning to distinguish between the presence and absence of persons and dogs in thermal images over time.

The second metric we used to evaluate the effectiveness of the proposed method is recall. Recall measures the proportion of actual positives that were correctly predicted, accounting for false negatives, which are cases that should have been flagged but were not. Recall is computed as

$$Recall=\frac{TP}{TP+FN}$$

The recall trend shown in Figure 6 mirrors the precision trend: both metrics increase with the number of epochs. This indicates an improving model performance over time. As shown in the plots, both precision and recall range from 0 to 1, demonstrating promising performance. Like precision, recall also exhibits a growing trend with an increasing number of epochs.

Precision and recall are frequently employed to gauge a model’s performance in classification tasks. Yet, to evaluate the model’s ability to precisely locate objects of interest within thermal images, we employed metrics like average precision (AP). AP assesses the accuracy of object detectors, such as the YOLO model employed in our study, by averaging precision values across recall values spanning from 0 to 1.

We aimed to compute the mean average precision (mAP), which entails using the Intersection over Union (IoU), precision, recall, precision–recall curve, and AP. Object detection models forecast both the bounding boxes and categories of objects within an image, with the IoU serving to ascertain the accuracy of these predictions.

The IOU indicates the extent of overlap between predicted and ground truth bounding boxes. An exact match results in an IOU of 1.0, while no overlap results in an IOU of 0.0. The IOU formula is

$$IOU=\frac{ao}{au}$$

where *ao* is the area of overlap, and *au* is the area of union.

In evaluating object detection models, the degree of overlap needed for successful recognition is defined by IOU thresholds. For example, mAP_0.5 measures accuracy at IOU = 50%, meaning detections with more than 50% overlap are considered successful. Higher IOU thresholds, like mAP_0.75, require more precise bounding boxes, making detection more challenging.

mAP is the average of AP values across all classes, and Figure 6 shows the mAP values for IOU = 50 (metrics/mAP_0.5) and for IOUs ranging from 50 to 95 (metrics/mAP_0.5:0.95), with a step size of 0.05.

Both metrics/mAP_0.5 and metrics/mAP_0.5:0.95 in Figure 6 exhibit increasing trends, indicating that the model effectively learns to locate humans and dogs in thermal images, correctly identifying the regions of interest.

In the following, we provide a detailed analysis related to each subplot.

With regard to the training losses, we comment on the following metrics:

- train/box_loss:
  - The box loss decreases steadily over the training epochs, indicating that the model has improving its ability to predict bounding box coordinates.
  - The smooth curve (dotted orange) aligns well with the actual results (solid blue), suggesting consistent improvement.
- train/seg_loss:
  - The segmentation loss also shows a steady decline, indicating the model’s improving performance in segmenting the lung regions.
  - Similar to the box loss, the smooth curve aligns with the actual results, reinforcing the trend in consistent improvement.
- train/cls_loss:
  - The classification loss starts high but decreases rapidly, showing the model’s quick adaptation in classifying lung regions correctly.
  - The smooth curve again aligns well with the actual results, supporting the trend in improving classification accuracy.
- train/dfl_loss:
  - The distribution focal loss (DFL) decreases over time, indicating better prediction of the bounding box distribution.
  - The alignment between the smooth and actual curves suggests a stable training process.

With regard to the validation losses, we comment on the following metrics:

- val/box_loss:
  - The box loss shows a downward trend with some fluctuations, which is typical in validation losses due to varying validation set difficulty.
  - The overall decreasing trend indicates the model generalizes well on the validation set.
- val/seg_loss:
  - The segmentation loss for validation follows a downward trend but with notable fluctuations. This could indicate varying difficulties in the validation samples or overfitting issues.
- val/cls_loss:
  - The classification loss shows a downward trend with fluctuations, indicating the model’s varying performance on different validation samples.
- val/dfl_loss:
  - The distribution focal loss decreases overall, similar to the other losses, but with fluctuations. This is consistent with the nature of validation losses.

With regard to the precision, recall, mAP50, and mAP50-95, we comment on the following metrics:

- metrics/precision(B) and metrics/recall(B):
  - The precision and recall for bounding box predictions (B) show an increasing trend, indicating better detection accuracy and recall over epochs.
  - Fluctuations are present, which is typical, but the overall trend is positive.
- metrics/precision(M) and metrics/recall(M):
  - The precision and recall for mask predictions (M) also show an increasing trend, indicating improving segmentation performance.
  - The fluctuations are present, but the overall improvement trend is clear.
- metrics/mAP50(B) and metrics/mAP50(M):
  - The mean average precision (mAP) at IoU threshold 50% shows improvement for both bounding boxes (B) and masks (M).
  - The increase is consistent, showing the model’s capability to accurately predict both bounding boxes and masks.
- metrics/mAP50-95(B) and metrics/mAP50-95(M):
  - The mAP across IoU thresholds from 50% to 95% for both bounding boxes (B) and masks (M) shows a steady improvement.
  - These metrics are more stringent and reflect the model’s robustness across various IoU thresholds.

To conclude this analysis, in the training phase, the model shows a consistent improvement in all loss metrics, indicating effective learning during training; in the validation phase the model generalizes well with a general downward trend in validation losses and improving performance metrics. Fluctuations in validation losses are typical and expected. The increasing trends in precision, recall, and mAP metrics suggest that the model is becoming more accurate and robust in both bounding box and mask predictions.

Overall, the training process appears to be effective, leading to a model that generalizes well on unseen data.

Table 2 shows the values obtained for bounding box precision, recall, mAP_0.5, and mAP_0.5:0.95 metrics (detailed for the single classes, i.e., nodule, cancer, and adenocarcinoma, and for all the classes).

In Table 2, it is evident that the bounding box precision and recall are 0.757 and 0.738, respectively, for all classes indicated under the all label. Moreover, we obtained a precision equal to 0.884 and a recall equal to 0.747 for the nodule label, a precision of 0.572 and a recall of 0.783 for the cancer label, and a precision of 0.815 and a recall of 0.685 for the adenocarcinoma label.

Table 3 shows the values obtained for the mask precision, recall, mAP_0.5, and mAP_0.5:0.95 metrics (detailed for the single classes, i.e., nodule, cancer, and adenocarcinoma, and for all the classes).

In Table 3, it is evident that the mask precision and recall are 0.75 and 0.733, respectively, for all classes indicated under the all label. Moreover, we obtained a precision equal to 0.865 and a recall equal to 0.73 for the nodule label, a precision of 0.572 and a recall of 0.783 for the cancer label, and a precision of 0.815 and a recall of 0.685 for the adenocarcinoma label.

The testing of the lung CT nodule instance segmentation model required, on average, 3.4 ms for preprocessing, 12.3 ms for inference, and 3.9 ms for postprocessing per image.

**Figure 7:**
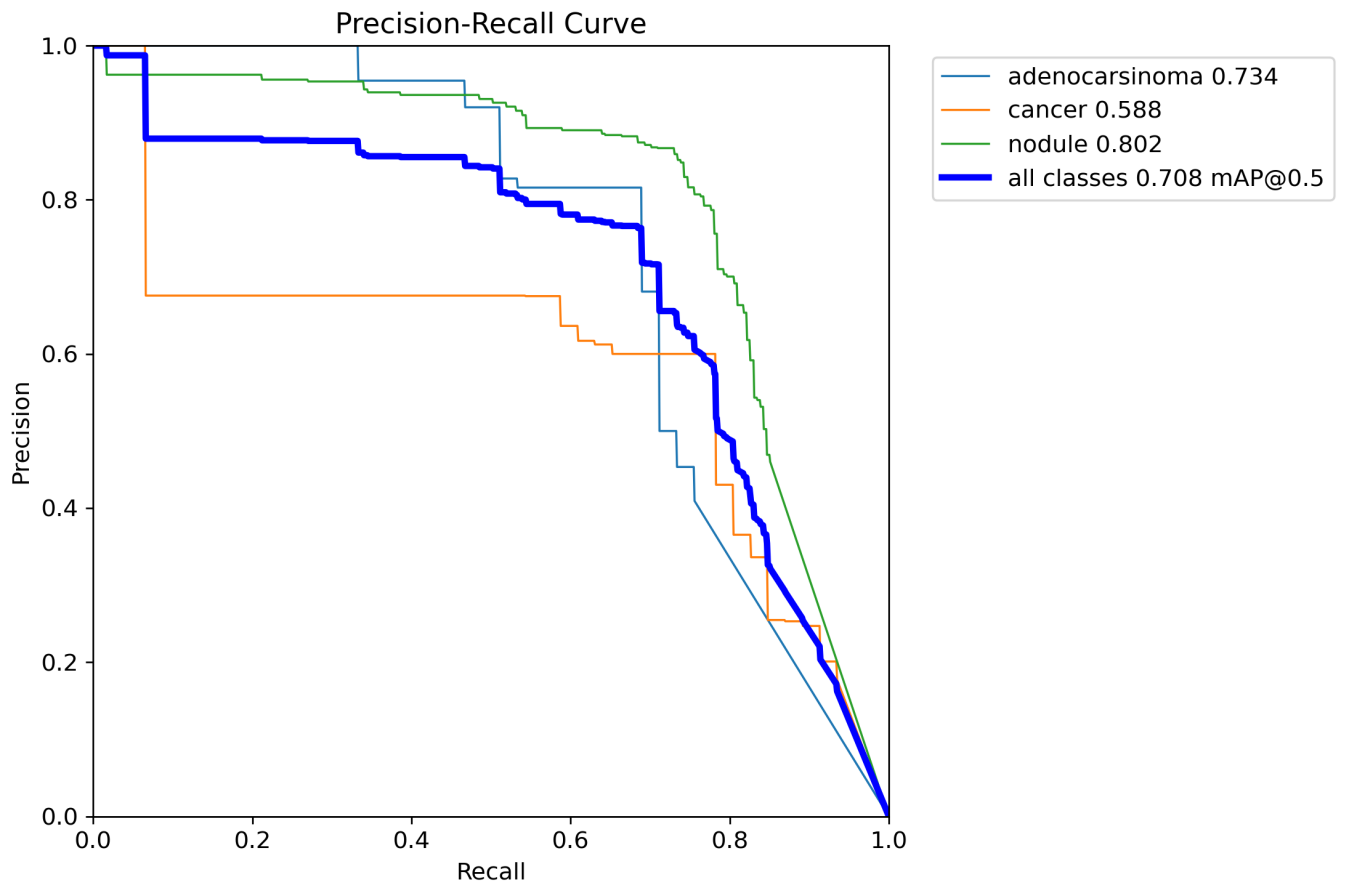
Precision–recall graph.

Moreover, with the aim to better evaluate the effectiveness of the proposed method, in Figure 7, we report the precision and recall values on a precision–recall graph.

The trend in this plot is expected to be monotonically decreasing due to the trade-off between precision and recall: increasing one typically decreases the other. Although the precision–recall graph is not always monotonically decreasing because of certain exceptions or data limitations, the plot in Figure 7 shows a decreasing trend for the labels involved.

The precision–recall plot also displays the Area Under the Curve (AUC) values for the involved classes (nodule, cancer, and adenocarcinoma) and the overall identification with mAP_0.5. As anticipated, the precision–recall trend is generally monotonically decreasing, as shown in the plot for all classes with mAP_0.5, which has an AUC of 0.940. This value is the average AUC for all considered classes. Specifically, the AUC for the adenocarcinoma class is 0.734; for the cancer class, it is equal to 0.588; and for the nodule one, it is equal to 0.802. Given that these metrics range from 0 to 1, these values indicate that the proposed model is particularly effective in detecting (generic) nodules and also adenocarcinoma, while it is less effective in the detection of cancer.

In the following, we provide interpretations of the curves:

- Adenocarcinoma (Blue Line):
  - The blue line represents the precision–recall relationship for the adenocarcinoma class.
  - With an average precision (AP) of 0.734, the model performs relatively well in detecting adenocarcinoma, maintaining high precision and recall values.
- Cancer (Orange Line):
  - The orange line represents the performance for the cancer class.
  - An AP of 0.588 indicates moderate performance, with a noticeable drop in precision as recall increases, suggesting that the model struggles more with this class compared to adenocarcinoma and nodules.
- Nodule (Green Line):
  - The green line shows the precision–recall for nodules.
  - With the highest AP of 0.802, the model performs best on this class, indicating high precision and recall across most thresholds.
- All Classes (Bold Blue Line):
  - The bold blue line represents the overall performance across all classes, with an mAP@0.5 of 0.708.
  - The mean average precision (mAP) at an IoU threshold of 0.5 provides a single metric summarizing the model’s performance across all classes.
  - An mAP@0.5 of 0.708 suggests that the model has a strong overall detection capability, balancing precision and recall well.

The model performs best on the nodule class, followed by adenocarcinoma, and then cancer, while the overall mAP@0.5 score of 0.708 shows that the model is quite effective in detecting the various classes.

**Figure 8:**
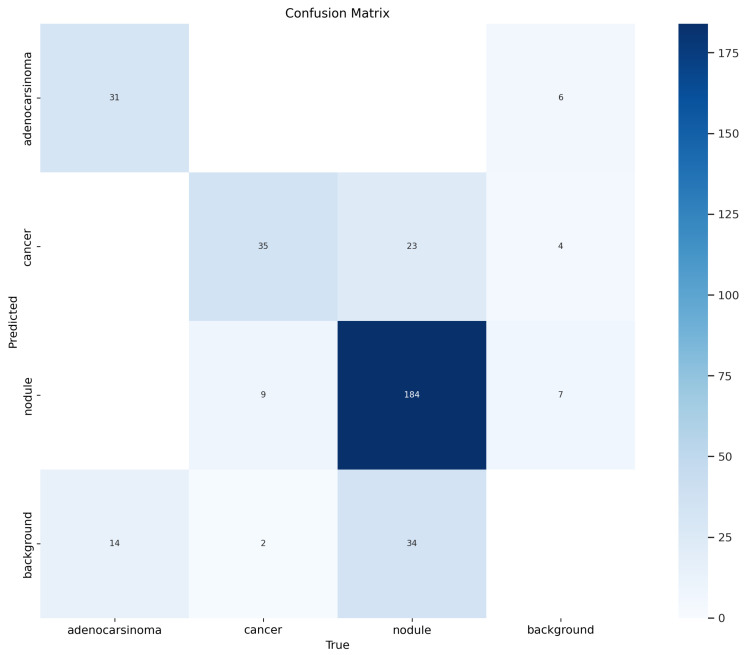
Confusion matrix.

Figure 8 shows a confusion matrix. A confusion matrix is a useful representation for evaluating the performance of a classification model, including object detection models like the YOLO one we exploited in this study for the instance segmentation task. In a nutshell, a confusion matrix provides a summary of the prediction results, showing the number of correct and incorrect predictions made by the model, categorized by the actual classes and predicted classes.

In the confusion matrix, the rows represent the actual labels (ground truth), while the columns represent the predicted labels by the model; in detail, we have the following:

- True Positives (TP): The number of times a class was correctly predicted.
- False Positives (FP): The number of times a class was incorrectly predicted.
- False Negatives (FN): The number of times the model failed to predict a class when it should have.
- True Negatives (TN): The number of times the background was correctly identified as not having an object.

Moreover, in object detection tasks, the “background” label plays a crucial role because it indicates areas where no objects of interest are present. This is essential for models like YOLO, which need to differentiate between objects and non-objects; in particular, we have the following:

- Background Row: This row contains the counts of predictions where the actual label is “background”. Ideally, the majority of these counts should fall in the “background” column, indicating that the model correctly identified non-object areas. Counts in other columns indicate false positives, where the model incorrectly identified the background as containing an object.
- Background Column: This column contains the counts of predictions that the model predicted “background”. Ideally, the majority of these counts should come from the “background” row, indicating correct predictions of non-object areas. Counts in other rows indicate false negatives, where the model failed to detect actual objects and mistakenly classified them as background.

In the confusion matrix in Figure 8, we can note that 31 adenocarcinomas were correctly marked as belonging to the right class, while 14 adenocarcinomas were wrongly marked as background. Relating to the cancer class, 35 cancers were correctly labeled as belonging to the right class, 9 cancers were wrongly labeled as nodule, and 2 cancers were wrongly marked as background. With regard to the nodule label, 184 nodules were rightly recognized as nodule, 23 nodules were wrongly detected as cancer, and 34 nodule were wrongly labeled as background.

**Figure 9:**
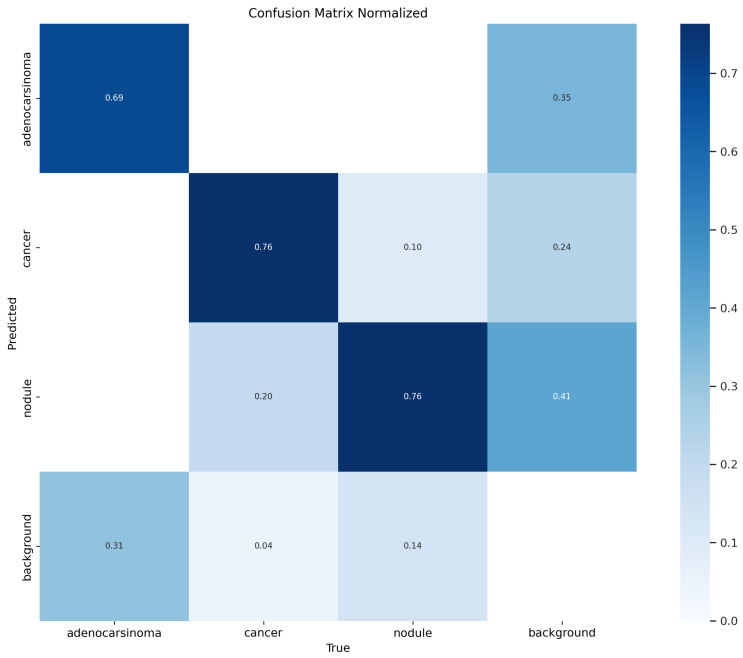
Normalized confusion matrix.

In Figure 9, we also show the normalized confusion matrix, where each value ranges from 0 to 1.

From the normalized confusion matrix, it is possible to immediately note that 69% of adenocarcinomas were rightly detected, and 76% of the cancers and nodules were also correctly marked as belonging to the relevant right category.

**Figure 10:**
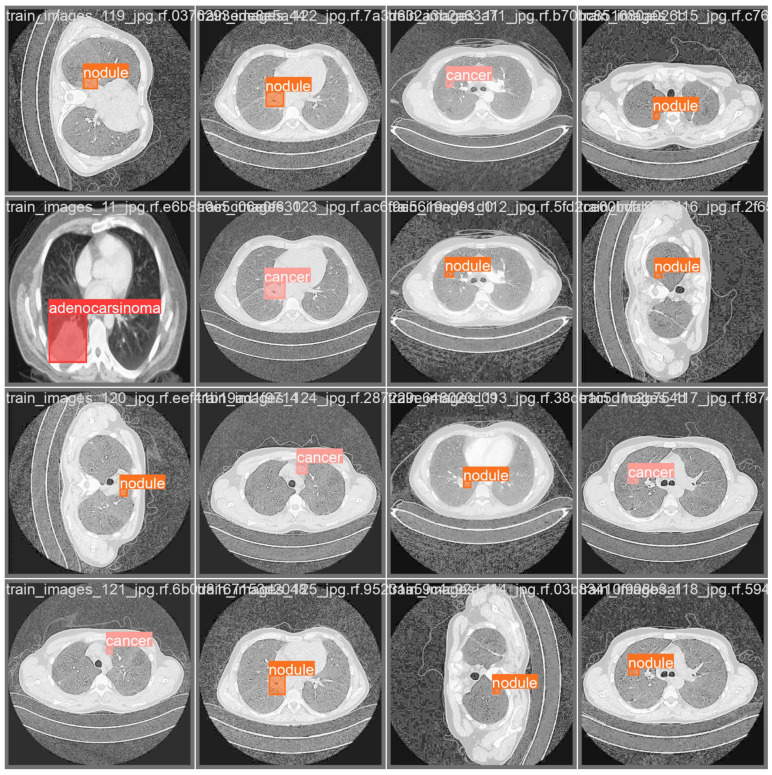
The results of the experimental analysis.

In Figure 10, we show several example of images belonging to the exploited lung CT dataset with the related detail about the segmentation annotation performed by expert radiologists.

In detail, in Figure 10, there are several images with different lung conditions: as a matter of fact, we can note instance segmentations related to nodules, cancer, and adenocarcinoma. It is possible to note that the segmented areas are decidedly small compared to the entire image; therefore, they are pathologies that may not always be immediately visible to the radiologist given the size of the masses in question. For this reason, the proposed method is of particular interest for screening.

**Figure 11:**
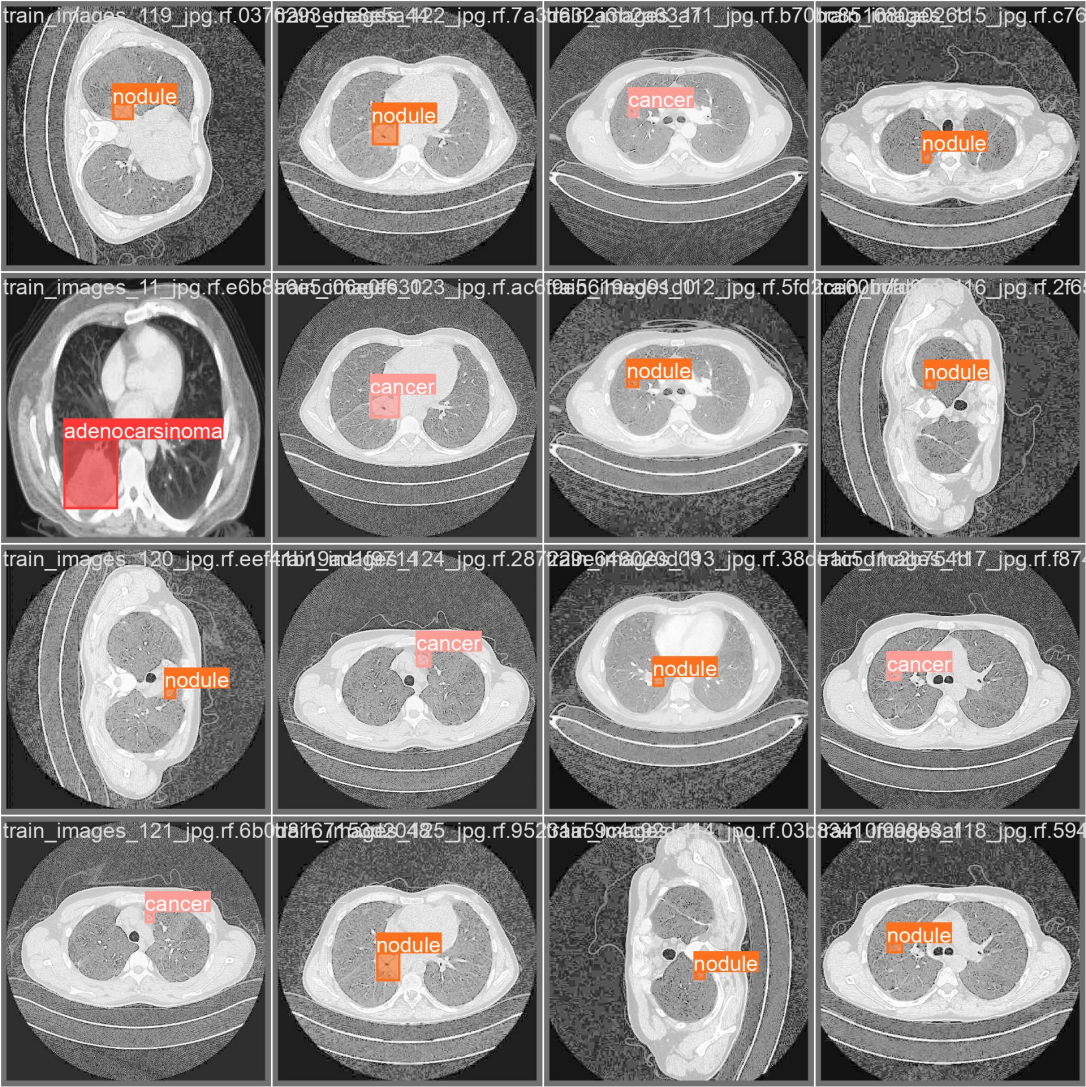
The results of the experimental analysis.

In Figure 11, we show the same set of images shown in Figure 10 with the segmentation and classification output performed by the trained model; in this figure, it is possible to directly compare the annotation segmentation with the prediction performed by the proposed model.

As shown by the lung CT images shown in Figure 11, we can note that in most cases, the proposed method is able to correctly predict the segmentation annotation and the related label. We note that in Figure 11, for each prediction, there is also the detection percentage related to the model’s confidence for a certain label assigned to the segmented area.

We note that the image related to adenocarcinoma was correctly predicted, with a percentage equal to 70%, while we also note that some masses labeled as cancer by radiologists were labeled as nodules; the same thing happened for some nodules labeled as cancer. We can note, however, that from a segmentation point of view, the proposed method is able to correctly label even extremely small masses, as can be seen in Figure 2, among other things even with very high classification percentages. We also note that in one image, two nodules were predicted, while in the same image, the radiologists highlighted only one nodule; cases like these, where the model highlights more masses than the radiologists, are very interesting and deserve a more detailed in-depth study aimed to understanding if there are actually more masses present in the lung CT. From this example, it can obviously be deduced that the model is also able to highlight multiple masses in a CT scan, even of different types. Finally, we also note that in two images, no masses were predicted by the proposed method, while in the same images, the radiologists had noted two nodules.

As shown in Figure 11, in the images in row 1, there are images showing high-confidence predictions for nodules (0.8 to 0.9), and in row 2, there are predicted nodules and cancers, similar to the ground truth (shown in Figure 10) but with slight variations in confidence scores. Images in row 3 include an adenocarcinoma prediction (0.7) that matches the ground truth, while images in row 4 show high-confidence predictions for nodules and cancers, corresponding closely with the ground truth labels (shown in Figure 10).

By comparing the ground truth images (shown in Figure 10) and the predictions with the related segmentation masks shown in Figure 11, we conclude that the predicted masks closely match the ground truth masks, indicating the model’s high accuracy in detecting nodules and cancers. Moreover, the confidence scores for predictions are generally close to those of the ground truth, demonstrating the reliability of the model. There are minor variations in the confidence scores between the ground truth and predictions. In some cases, the exact positioning of the bounding boxes might differ slightly, but the identified regions largely overlap. The predictions consistently identify the same types of abnormalities (nodules, cancers) as the ground truth. The model accurately identifies regions labeled as adenocarcinoma, indicating its effectiveness in distinguishing different types of abnormalities.

The proposed instance segmentation model demonstrates high performance in segmenting lung nodules and cancers, as evidenced by the close alignment between the ground truth and predicted labels. The minor differences in confidence scores and bounding box positions do not significantly impact the overall accuracy and reliability of the model predictions.

## 5. Conclusions and Future Work

Considering the importance of screening for the detection of abnormal masses in lung, in this paper, we propose a method aimed to automatically detect and locate masses in lung CT, in particular the proposed method is able to segment (i.e., divide an image into segments, each of which represents a separate object or part of the image) a lung CT under analysis with masses classified as (generic) nodule, cancer, or adenocarcinoma. For model training, we resorted to the YOLO instance segmentation model, one of the most widespread models exploited for real-time segmentation. To evaluate the effectiveness of the proposed method, a dataset composed of 3654 lung CT images was considered, obtaining an average precision and recall, respectively, equal to 0.757 and 0.738 in the classification task. Furthermore, we obtained an average mask precision and mask recall equal to 0.75 and 0.733, which indicates that the proposed method is able to not only classify masses in nodule, cancer, and adenocarcinoma but also segment the areas, thus performing an effective instance segmentation.

While YOLOv8’s adaptation for instance segmentation brings several advantages, there are also some inherent limitations and challenges associated with this approach; for example, the addition of a mask prediction head and the need for detailed segmentation increase the computational and memory requirements compared to standard object detection tasks. This can limit model deployment on resource-constrained devices. Furthermore, segmentation tasks are inherently more complex and computationally intensive, leading to longer inference times compared to pure object detection. As a matter of fact, while YOLOv8 is designed for real-time applications, adding segmentation capabilities can introduce a trade-off between accuracy and speed. Ensuring both high segmentation accuracy and real-time performance is challenging. Segmenting very small objects remains challenging, as the feature maps may not have enough resolution to accurately delineate tiny details. Accurately segmenting overlapping objects can be problematic, as the model might struggle to separate closely packed instances within the same class. The predicted masks can sometimes be coarse and less detailed, especially for objects with intricate shapes.

To address these limitations, several strategies can be employed; for instance, it is possible to use model compression techniques like pruning and quantization to reduce the computational burden, enhance the training dataset with extensive data augmentation to improve generalization and performance, and employ advanced postprocessing techniques to refine segmentation results and improve mask quality.

As future work, we plan to consider additional model, for instance, other YOLO model versions, such as versions 9 and 10 (we experimented with the eight one in this study). Moreover, we plan to take into account the so-called explainability, i.e., a way to understand which area of the segmented images from the model’s point of view most influenced the segmentation. As a matter of fact, we plan to investigate newer versions of the YOLO model, which are likely to incorporate further innovations in neural network architecture and training methodologies, which be essential for maintaining state-of-the-art performance in object detection and instance segmentation tasks. Furthermore, integrating explainability techniques into YOLO models will be crucial for enhancing transparency and trustworthiness, allowing researchers and practitioners to better understand the decision-making processes of these models. These efforts will provide a clearer road map for advancing this research, paving the way for more robust, interpretable, and efficient computer vision systems.

## Figures and Tables

**Table 1:** Comparison of state-of-the-art segmentation methods using deep learning.

Method	Dataset	References
U-Net	LUNA16	[43]
FCN + Nodule Detection	LUNA16	[44]
3D U-Net	LUNA16	[45]
Self-Supervised Learning	LIDC-IDRI	[46]
CNN + CRFs	JSRT	[47]
Attention-Guided U-Net	COVID-19-CT-Seg	[48]
Dual-Branch Network	COVID-19-CT-Seg	[49]
Transformer-Based Network	LUNA16, COVID-19-CT-Seg	[50]
Cross-Attention Network	COVID-19-CT-Seg	[51]
GANs	LUNA16, LIDC-IDRI	[52]

**Table 2:** Bounding Box Classification results.

Class	Image	Instances	Box (P)	Box (R)	Box (mAP50)	Box (mAP50-95)
all	324	332	0.757	0.738	0.71	0.488
adenocarsinoma	324	45	0.815	0.685	0.713	0.492
cancer	324	46	0.572	0.783	0.588	0.467
nodule	324	241	0.884	0.747	0.831	0.505

**Table 3:** Mask Classification results.

Class	Image	Instances	M (P)	M (R)	M (mAP50)	M (mAP50-95)
all	324	332	0.75	0.733	0.708	0.438
adenocarsinoma	324	45	0.815	0.685	0.734	0.539
cancer	324	46	0.572	0.783	0.588	0.366
nodule	324	241	0.865	0.73	0.802	0.41

## Data Availability

The dataset (i.e., images and annotation segmentations) is freely available for research purposes at the following URL: https://universe.roboflow.com/varun-18tlk/lung-nodule-segmentation-study/dataset/3, accessed on 19 June 2024.
